# Genetic Manipulation of NK Cells for Cancer Immunotherapy: Techniques and Clinical Implications

**DOI:** 10.3389/fimmu.2015.00266

**Published:** 2015-06-10

**Authors:** Mattias Carlsten, Richard W. Childs

**Affiliations:** ^1^Hematology Branch, National Heart, Lung and Blood Institute, National Institutes of Health, Bethesda, MD, USA

**Keywords:** NK cells, genetic manipulation, viral transduction, electroporation, cancer immunotherapy

## Abstract

Given their rapid and efficient capacity to recognize and kill tumor cells, natural killer (NK) cells represent a unique immune cell to genetically reprogram in an effort to improve the outcome of cell-based cancer immunotherapy. However, technical and biological challenges associated with gene delivery into NK cells have significantly tempered this approach. Recent advances in viral transduction and electroporation have now allowed detailed characterization of genetically modified NK cells and provided a better understanding for how these cells can be utilized in the clinic to optimize their capacity to induce tumor regression *in vivo*. Improving NK cell persistence *in vivo* via autocrine IL-2 and IL-15 stimulation, enhancing tumor targeting by silencing inhibitory NK cell receptors such as NKG2A, and redirecting tumor killing via chimeric antigen receptors, all represent approaches that hold promise in preclinical studies. This review focuses on available methods for genetic reprograming of NK cells and the advantages and challenges associated with each method. It also gives an overview of strategies for genetic reprograming of NK cells that have been evaluated to date and an outlook on how these strategies may be best utilized in clinical protocols. With the recent advances in our understanding of the complex biological networks that regulate the ability of NK cells to target and kill tumors *in vivo*, we foresee genetic engineering as an obligatory pathway required to exploit the full potential of NK-cell based immunotherapy in the clinic.

## Introduction

Natural killer (NK) cells are immune cells primarily found in the blood, liver, spleen, bone marrow, and to a lesser extent, in lymph nodes (1). They were initially identified based on their ability to lyse tumor cells without a need for priming (2–5). NK cells are now known to play an important role in host immunity against both cancers and certain viral infections (6–8).

NK cells can mediate cytotoxicity via multiple distinct mechanisms. Degranulation is the most studied cytotoxicity pathway, where NK cells release cytotoxic granules upon contact with the target. Cytotoxicity via this pathway is dictated by a balance of signals from an array of germline encoded activation and inhibitory cell surface receptors. Most activation receptors need simultaneous co-stimulation by other activation receptors to trigger NK cell cytotoxicity (9). One exception from this rule is the Fc receptor CD16, which alone can trigger NK cell degranulation against antibody-coated target cells via antibody-dependent cellular cytotoxicity (ADCC) (9). Other routes by which NK cells can kill targets are the death receptor pathways TRAIL/TRAIL-R and Fas/FasL. Instead of triggering release of cytotoxic granules, death receptor pathways prompt apoptosis via caspase activation in target cells.

More than a decade has passed since initial reports established the anti-tumor potential of NK cells in patients with cancer. These studies showed that haplo-identical donor NK cells could prevent relapse in acute myeloid leukemia (AML) following hematopoietic stem cell transplantation (HSCT) and that adoptively infused mature donor NK cells could induce remission in AML patients (6, 10). Despite this revelation, doubts remain about the true therapeutic potential of NK cells in cancer immunotherapy. In contrast to therapy utilizing T cells, enthusiasm for NK cell-based immunotherapy has been tempered by uncertainties about their *in vivo* persistence, and doubts regarding their ability to migrate to tumor tissues following adoptive infusions. Although recent data have shown CMV reactivation reduces the risk for AML relapse following HSCT (11) potentially caused by CMV-induced NK cells cross-reacting with AML cells, NK cells, unlike T-cells, lack antigen specificity, further tempering enthusiasm for their use as immune effectors in cellular therapy.

Genetic manipulation of NK cells to improve their persistence, cytotoxicity, tumor targeting capacity, and ability to home to disease sites *in vivo* holds potential to advance the efficacy of NK cell-based cancer immunotherapy. However, until relatively recently, the genetic manipulation of NK cells has proven to be challenging. Viral transduction, successfully used for T cells, has been associated with low levels of transgene expression and unfavorable effects on cell viability when used with NK cells. Recent optimization of viral transduction and the establishment of electroporation technologies for efficient gene transfection have revived the enthusiasm for studies evaluating genetic modification of NK cells. Investigators around the world are now exploring the potential of multiple different NK cell modalities to genetically reprogram with the overall aim of further improving upon their capacity to kill tumors in cancer patients. One example of how this technique can be utilized is to introduce genes into NK cells coding for gamma-cytokines (IL-2 and IL-15) to induce independence from the obligate need of exogenous cytokines for proper *in vivo* persistence and expansion post infusion. This and similar strategies may further improve the efficacy of NK cell-based immunotherapy, as tumor regression following adoptive NK cell infusions in AML patients has been reported to be dependent on their ability to expand *in vivo* (6), while being limited by regulatory T cells also mobilized following exogenous cytokine administration (12, 13). The introduction of chimeric antigen receptors (CARs) and the down-regulation of inhibitory NK cell receptors such as NKG2A are additional examples of specific genetic manipulations that can be utilized to improve the outcome of adoptive NK cell immunotherapy.

Given their rapid and efficient method of recognizing tumor cells, NK cells represent a unique immune cell to genetically reprogram in an effort to improve the outcome of cell-based cancer immunotherapy. This review focuses on methods for introducing transgenes into NK cells and the advantages and limitations of such strategies. It also gives an overview of strategies for genetic reprograming of NK cells that have been evaluated to date and an outlook on how these specific strategies may be best utilized in clinic to maximize the anti-tumor potential of NK-cell based immunotherapy.

## Methods and Challenges with Genetic Manipulation of NK Cells: Viral Transduction Versus Transfection

Genetic manipulation of T cells has successfully been used in both preclinical and clinical research (14). In contrast, studies on genetically engineered NK cells have historically been limited by poor efficacy of transgene delivery and substantial procedure-associated NK cell apoptosis. In this section, we discuss available approaches for gene delivery into NK cells, characterizing how each approach developed over time while highlighting the positive and negative aspects of each method (Box 1).

Box 1 **Pros and Cons for Methods of Genetic Modification of NK Cells.**

**Table:** 

Method	Pros	Cons	Vector/apparatus used
Viral transduction	Stable transgene expression	Risk for sustained and uncontrollable adverse events due to stable transgene expression	Retroviral vectors
			Lentiviral vectors
			Adenoviral vectors
			Vaccinia virus vectors
	Well characterized when used with other immune cells (e.g., T cells)	Risk for insertional mutagenesis and immunogenicity	
	Can be used with gene editing technologies, such as CRISPR	Cellular enrichment may be needed and viability may be compromised	

Transfection	High transduction efficiency without compromising viability	Transient transgene expression may not be sufficient to induce long-term clinical responses	Amaxa
	Viral vector independent; less regulatory issues; no need for high-level biosafety laboratory		BioRad
	Can be used with gene editing technologies, such as CRISPR		BTX
			MaxCyte

### Viral transduction

The reduced efficacy of viral transduction of NK cells compared to T cells may in part be related to the innate properties that characterize NK cells. Innate immune receptors, such as pattern recognition receptors that recognize foreign genomic material, are likely involved in triggering apoptosis of NK cells following viral transduction (15). Best results from studies of viral transduction of NK cells have been achieved using either NK cell lines or primary NK cells that have undergone expansion *ex vivo* (Table 1). In contrast, viral transduction of primary resting human NK cells typically results in substantially lower transduction efficiencies. Most studies on viral transduction of NK cells have utilized retro- and lentiviral vectors. Although adenoviral- and vaccinia virus vectors have been utilized for transduction of NK cells, their use has been limited and they will not be discussed further in this review.

**Table 1:** **Overview of techniques used to genetically modify NK cells with reported gene delivery efficacies and effect on cell viability**.^a^

Method		NK cell source	Efficacy (%)	Viability	Reference
Viral transduction	Retroviral vector	NK cell lines	1–10	n.r.	(16–19)
		Resting/short-term activated NK cells	6–50	n.r.	(18)
		Expanded NK cells	6–96	n.r.	(20–25)
	Lentiviral vector	NK cell lines	2–97	n.r.	(26–29)
		Resting/short-term activated NK cells	3–73	n.r.	(15, 26, 28, 30, 31)
		Expanded NK cells	90	95%	(26)
Transfection	Nucleofection (RNA and DNA)	NK cell lines	17–48	45–97%	(32–35)
		Resting/short-term activated NK cells	11	n.r.	(33)
		Expanded NK cells	–	–	–
	Electroporation (RNA and DNA)	NK cell lines	1–80	90%	(36–39)
		Resting/short-term activated NK cells	40–90	86–90%	(40, 41)
		Expanded NK cells	61–81	89–90%	(40, 41)

*^a^Only those studies that have reported transgene expression following genetic manipulation of NK cell are reported in this table*.

*n.r., not reported*.

*Retroviral vectors* were the first viral vectors used to genetically modify NK cells. The first report on retroviral transduction of NK cells was published in the late 1990s and focused on genetic manipulation of the NK cell line NK-92 (16). This study reported a transduction efficacy of only 2–3%. Optimization of retroviral transduction approaches over the past decade has resulted in higher transduction efficiencies, especially when used with human NK cells that have undergone *ex vivo* expansion (Table 1). A recent report showed that retroviral transduction of *ex vivo* expanded NK cells with genes coding for either IL-15 or membrane bound IL-15 (mbIL-15) resulted in an average 69 and 71% transduction efficiency, respectively (25). Although retroviral transduction of NK cells has been reported to not alter the function, phenotype, and proliferative capacity of NK cells (20, 23), their viability following retroviral transduction has rarely been reported. A significant deleterious impact on the viability of primary NK cells undergoing retroviral transduction may preclude utilizing this approach in a clinical setting. Further, retroviral transduction also requires active cell division, impeding the use of this method with primary non-activated NK cells. This limitation is less important when retroviral transduction is utilized with NK cell lines such as NK-92 that have continuous and unlimited proliferation capacity. However, as discussed later in this review, it is important to note that this NK cell line does have phenotypic and function differences from primary human NK cells, which may have therapeutic implications for clinical therapy.

#### Lentiviral Vectors

More recently, studies evaluating transduction of NK cells using lentiviral vectors have been pursued. In contrast to transduction with retro- and adenoviral vectors, lentiviral vectors can incorporate transgenes into the genome of non-dividing cells. Further, lentiviral vectors allow for gene modification of NK cells without altering their phenotypic and functional properties as occurs following stimulation with i.e., cytokines. The first report on the successful use of lentiviral vectors for genetic modification of NK cells was performed in primary murine NK cells (42), with subsequent studies establishing that lentiviral transduction of human NK cells could also be achieved (Table 1). Although most studies have reported lentiviral transduction of NK cell lines with efficiencies of 15–40% (27, 28), the efficiency highly varies from only a few percent to nearly 100%, and in some cases, multiple rounds of transduction are required (26, 29). Recent data indicate that transduction efficiencies of primary human NK cells can be increased by drug-induced inhibition of intracellular innate immune receptors in NK cells (15). Unfortunately, and similar to studies utilizing retroviral transduction, the viability of NK cells after lentiviral transduction has rarely been reported. Using an optimized protocol, our lab has achieved a maximum transgene expression in up to 60% of *ex vivo* expanded NK cells 3 days after lentiviral transduction with GFP without incurring any deleterious effects on NK cell viability, phenotype, or function (Personal communication, R. Childs).

In summary, viral transduction of NK cells results in variable transduction efficacies and may require multiple rounds of transduction and/or post transduction cell enrichment to achieve acceptable transgene expression. Further, viral associated cell death and the need for post-transduction enrichment may compromise the clinical utility of this approach. Finally, although the risk may be low, the possibility of viral-induced insertional mutagenesis and immunogenicity (43, 44) occurring post transduction must be considered when utilizing this methodology in the clinic. Nevertheless, viral transduction of NK cells does achieve stable transgene expression which, depending on how the NK cell is being genetically modified, might be required to induce a durable and long-term clinical response.

### Transfection

Compared to viral transduction, transfection of NK cells appears to be associated with lower degrees of apoptosis, less inter-individual and inter-experimental variability, with transgene delivery efficiency being completely independent of cellular division. In most cases, this approach results in a more rapid albeit transient expression of the transgene as compared to viral transduction where genes must first be incorporated into the cellular genome before expression can occur. Gene transfer using transfection can be achieved by either *electroporation* (including *nucleofection*) or *lipofection*. Since the latter has been used only in a few studies (45), this review will focus on strategies utilizing the electroporation approach.

Electroporation is a method where genetic material is delivered into cells following a short electric pulse that temporarily induces small pores in the cell membrane, allowing charged molecules such as DNA and RNA to move into the cell. This technology was first used with NK cell lines in the late 1990s (32, 36–38, 46, 47) and more recently has been used to genetically manipulate primary NK cells to express CARs (35, 39, 48) or cytokines for autocrine growth stimulation (49). With technological advances and the use of mRNA instead of cDNA, transfection efficiencies have increased dramatically, reaching up to 90% or more while having only a minimal deleterious effect on cell viability (Table 1). Remarkably, using mRNA electroporation, transfection efficiencies of 80–90% can be achieved in not only *ex vivo* expanded cells but also in primary resting (non-cytokine activated) human NK cells (40). Despite this remarkable advance, a detailed characterization on the effects of electroporation on the phenotype, function, and proliferative capacity of NK cells following electroporation has yet to be published.

As electroporation does not involve viral vectors, its use in the preclinical and clinical setting is associated with less regulatory issues. Also, as indicated above, electroporation most often leads to transient transgene expression, which may be viewed favorably from a safety perspective when new transgenes with unknown potential toxicities are being explored in early clinical trials. Regimens that use DNA electroporation technology have been employed to generate stable transgene expressing cells. Although the efficacy of this approach is typically lower than that achieved with viral transduction, it may be improved if combined with targeted integration techniques that avoid random integration in inactive heterochromatin regions. Such strategies also reduce the risk for off-target effects, including gene silencing due to random integration in active genes and integration in hot-spots that may trigger malignant transformation. With advantages in design of guiding RNAs and by having better on-target specificity compared to other gene editing technologies such as ­Zink-Finger nuclease (ZFN) and the transcription activator-like effector nucleases (TALEN) technologies, the recently developed clustered regularly interspaced short palindromic repeats (CRISPR) technique has rapidly become a popular tool for targeted gene integration (50). The CRISPR/Cas9 system induces permanent modifications at specific sites of the genome via double-strand breaks (DSBs), and can be used to integrate new genes at specific sites via homology-directed recombination (50). Although only moderate degrees of genome integration are currently being achieved with this technique today, the CRISPR/Cas9 system could be used to produce stably transduced NK cells by gene editing of primary NK cells prior to their *ex vivo* expansion.

## Gene Modification Strategies Aimed at Improving the Efficacy of NK Cell-Based Cancer Immunotherapy

**Figure 1:**
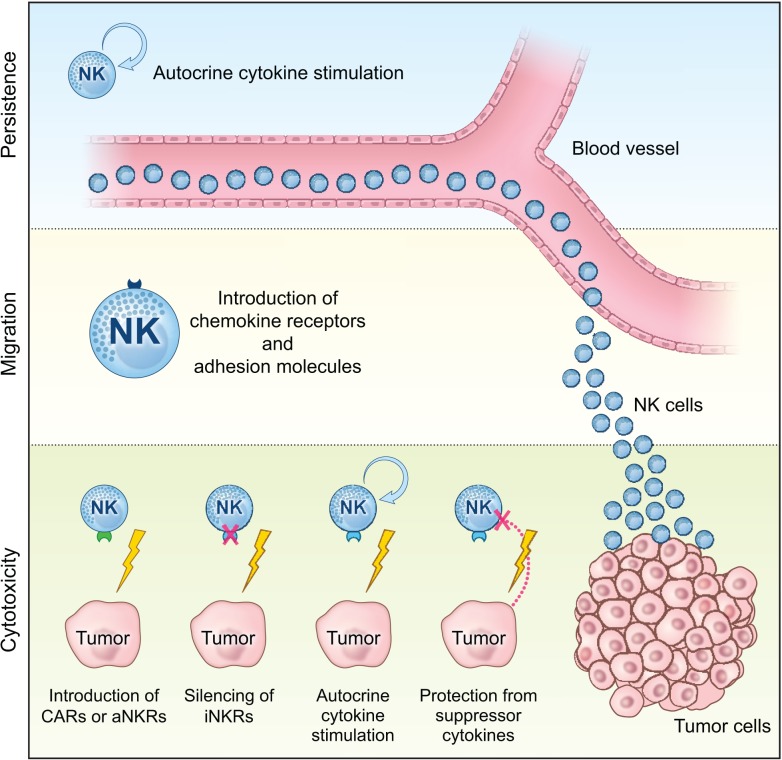
**Schematic overview of how genetic manipulation can be can be used to improve the efficacy of NK cell-based cancer immunotherapy in the clinic**. Genetic engineering of NK cells to promote persistence and expansion by autocrine cytokine stimulation, migration to the tumor tissue via introduction of receptors involved in cellular homing (i.e., chemokine receptors and adhesion molecules), as well as bolstering their anti-tumor cytotoxicity via introduction of CARs or activating NK cell receptors (aNKRs) or via silencing of inhibitory NK cell receptors (iNKRs), protection from suppressive cytokines in the tumor environment, and boosted function via autocrine cytokine stimulation.

With new advances in the field, genetic manipulation of NK cells has opened up possibilities to study many different pathways involved in NK cell tumor targeting and the ability to genetically modify NK cells to improve their tumor cytotoxicity. Here, we will discuss reported gene modification strategies that can improve *in vivo* persistence and expansion, tumor tissue migration, and the tumor targeting capacity of adoptively infused NK cells (Figure 1, Table 2 and Box 2).

**Table 2:** **Overview of strategies evaluated for improving the anti-tumor efficacy of primary human NK cells and NK cell lines *in vitro* and in preclinical animal models**.

Modality	Strategy	Molecule	Method	Reference
Persistence/expansion	Cytokine stimulation	IL-2	RV	(16, 51)
		IL-15/mbIL-15	RV, EP, LV	(25, 29, 49, 52)
Migration	–	–	–	–
Cytotoxicity	Redirected targeting	αCD19 CAR	RV, LV, EP	(28, 39–41, 53)
		αCD20 CAR	RV, LV, EP	(28, 53, 54)
		αCD33 CAR	EP	(38)
		αCD138 CAR	LV	(24, 48)
		αCS1 CAR	LV	(55)
		αGD2 CAR	RV	(23, 56)
		αHER2 CAR	RV, EP	(22, 57)
		αerbB2 CAR	EP	(35)
		αCEA CAR	EP	(36)
		αEpCAM CAR	LV	(29)
		αNKG2D-L CAR	RV	(58)
		αTRAIL-R1 CAR	RV	(58)
		αGPA7	RV	(19, 59)
	ADCC	HA-CD16	RV	(19)
	Cytokine stimulation	IL-2	RV	(16)
		IL-15/mbIL-15	RV, LV, EP	(25, 29, 49, 52)
	Protection from suppressive cytokines	DNTβRII	EP	(34)
	Receptor silencing	NKG2A (shRNA)	LV	(31)

*RV, retroviral transduction; LV, lentiviral transduction; EP, electroporation; ADCC, antibody-dependent cellular cytotoxicity; HA-CD16, high-affinity CD16; DNTβRII, double negative TGF-β RII*.

Box 2 **Examples of NK Cell Modalities to Gene Manipulate for Improved Clinical Efficacy.**

**Table:** 

Persistence/expansion	Autocrine cytokine production (IL-2, IL-15, and mbIL-15)
Migration	CCR7 and CXCR3
Cytotoxicity	CARs, CD16, autocrine cytokine production (IL-2 and IL-15), and overexpression of double negative TGF-β II receptor to avoid suppressive effects of TGF-β. Silencing of inhibitory NK cell receptors, such as NKG2A

### Strategies to improve persistence and expansion of infused NK cells

*In vivo* persistence and expansion of infused NK cells have been shown critical for inducing tumor regression following adoptive NK cell infusion (6). Using retroviral transduction of the IL-2 gene into NK-92 cells, Nagashima et al. were able to propagate this NK cell line for up to 5 months *in vitro* without the addition of exogenous cytokines (16). Further, IL-2-expressing NK-92 cells where shown to also have enhanced tumor cytotoxicity compared to non-transduced parental NK-92 cells that were stimulated with exogenous IL-2. In line with these *in vitro* findings, these genetically modified cells showed improved *in vivo* persistence and anti-tumor responses when infused into tumor-bearing mice. Similar data with IL-2 gene delivery in expanded NK cells were reported by Konstantinidis et al. (51). As observed with IL-2 transduced NK-92 cells, retroviral transduction of *ex vivo* expanded NK cells with the mbIL15 gene also dramatically increased their survival *in vitro*; median cell recovery was 85% for mbIL-15 NK cells after 7 days in culture without IL-2, whereas mock-transduced NK cells were essentially undetectable (25). Hence, the strategy of introducing genes coding for gamma-cytokines to improve *in vivo* NK cell persistence and expansion following infusion independent of exogenous cytokine administration appears promising.

### Strategies to enhance migration of infused NK cells

Proper tumor tissue homing of infused NK cells is a prerequisite for their ability to induce tumor regression. However, studies characterizing the *in vivo* migration capacity of adoptively infused NK cells have been largely overlooked (60). Recent evidence suggests non-expanded and expanded NK cells have different migration patterns when infused into animal models (61). Moreover, using trogocytosis to transfer premade cell surface molecules from a feeder cell line to NK cells, Somanshi et al. have shown that migration of infused NK cells can be redirected by equipping them with the lymph node homing receptor CCR7 (62). Despite these data, no study has so far used gene modification techniques to actively direct infused NK cells to selected organs. Based on data from Somanshi et al., we have been able to use mRNA transfection to genetically engineer NK cells with the CCR7 receptor to improve their migration toward one of its ligands CCL19 (Carlsten M., Manuscript in preparation, April 2015). Other strategies may involve utilizing chemokine receptors, such as CXCR3 to improve NK cell migration to inflamed tissues, such as those infiltrated with metastatic tumors (63).

### Strategies to increase tumor cytotoxicity by infused NK cells

The majority of reports on expression of transgenes in NK cells have characterized the effects of CARs in NK cell lines, expanded NK cells, and primary non-expanded NK cells (Table 2). CARs are engineered receptors that have the extracellular specificity of an antibody combined with potent intracellular signaling adaptors such as CD3ζ, CD28 and/or 4-1BB. Importantly, these receptors do not require stimulation through co-receptors to trigger robust anti-tumor cytotoxicity. The recent breakthrough success of anti-CD19 CAR T cell therapy in patients with B cell malignancies has stimulated the research community to develop and investigate a wide array of CARs against multiple different epitopes expressed on numerous tumor types (64). Several of these CARs have been explored in NK cells (Table 2). CD19 and CD20 specific CARs against B cell malignancies (39–41, 53, 54), and CARs targeting CD33 on leukemia cells (38), CS1 and CD138 on myeloma cells (24, 48, 55), GD2 on neuroblastoma cells (23, 56), Her2/Neu and erbB2 on breast cancer cells (22, 35), carcinoembryonic antigen (CEA) on colon cancers (36), EpCAM on epithelial tumors (29), GPA7 on melanoma (59), NKG2D ligand on leukemia and solid tumors, and TRAIL-R1 on various tumor targets (58) have all been shown to have the capacity to redirect NK cell cytotoxicity against their target antigens. The majority of these studies have used viral vectors to transduce CARs into the NK cell, albeit electroporation has also been used in a few studies (Table 2).

Based on clinical data showing superior response rates in rituximab-treated lymphoma patients homozygous for the high-affinity CD16-158V polymorphism (HA-CD16) compared to those who carry the low-affinity CD16-158F (LA-CD16) polymorphism (65, 66), several groups have recently addressed whether introduction of the HA-CD16 gene into NK cells lacking this polymorphism can be used as a strategy to augment ADCC against tumors. This approach has appeal as only a minority of patients is homozygous for HA-CD16 (67). Moreover, in contrast to CAR NK cells, infusions of NK cells genetically modified to express HA-CD16 may be used to improve the outcome of virtually any malignancy for which there is an FDA approved IgG1 antibody, without the expectation for any severe off target side-effects. *In vitro* experiments conducted by Binyamin and colleagues showed significantly improved cytotoxicity against a rituximab-coated B lymphoma cell line following stable transduction of the CD16 negative NK-92 cell line with HA-CD16 compared to NK-92 cells were equipped with LA-CD16 (19). Recently, our group explored a similar approach, where *ex vivo* expanded NK cells from CD16-158F/F (LA-CD16) donors were found to have substantially augmented ADCC following electroporation with mRNA coding for the HA-CD16 (68). These data suggest the addition of the HA-CD16 gene to patient NK cells that already express endogenous CD16 can be used to augment their ability to induce ADCC, and that this approach could be used as a strategy to improve the efficacy of antibody-based therapies for cancer patients.

Introduction of genes that render NK cells insensitive to suppressive cytokines such as TGF-β, thereby preserving their cytotoxicity, has also been studied. Yang et al. generated an NK-92 cell line resistant to the suppressive effects of TGF-β by genetically modifying them to express the dominant negative mutant form of TGF-β type II receptor (DNTβRII) on their surface (34). Adoptive transfer of these TGF-β insensitive NK-92 cells in lung cancer-bearing mice was associated with increased levels of IFN-γ released from the infused cells and resulted in increased survival rates compared to mice treated with wild-type NK-92.

Genetic reprograming of NK cells may also be directed to achieve specific protein silencing with the aim of improving tumor targeting by circumventing NK cell inhibitory signals induced upon interaction with tumor cells. Initial studies have focused on the use of shRNA technology for this purpose. In this context, shRNAs expressed inside cells are processed by the Dicer endonuclease complex to generate double-stranded small interfering RNAs that prevent translation of their target mRNAs (69), shRNAs have been used successfully to knock-down expression of the HLA-E-binding inhibitory NK cell receptor NKG2A (31). Using an inducible vector in IL-2 activated NK cells, Figueiredo et al. observed a 40% increased killing capacity against the HLA-E expressing cell line K562 HLA-E. Using a similar approach with the NK cell line NKL, our group observed increased killing capacity of HLA-E expressing 721.221 cells *in vitro* and in a preclinical mouse model (70). Further details on protocols for shRNA-mediated protein silencing in NK cells can be found in Purdy et al. (71). To date, studies utilizing CRISPR, ZFN, or TALEN to genetically modify NK cells to silence their inhibitory receptors for the same purpose of enhancing the anti-tumor capacity of NK cells have not yet been reported.

In conclusion, an array of gene modification strategies for NK cells has now been reported. Several of them hold promise for improving clinical responses of NK cell-based cancer immunotherapy. However, to date, few have been translated into clinical studies. The following section will discuss how these strategies can be incorporated in clinical NK cell cancer immunotherapy.

### Considerations for the Development of Clinical Protocols using Genetically Engineered NK Cells

Challenges associated with genetic manipulation of NK cells have significantly delayed the debut of this strategy in clinical cancer therapy. While recently initiated trials (NCT00995137 and NCT01974479) exploiting the role for CAR19-expressing *ex vivo* expanded NK cells in patients with B cell malignancies will give us a first insight into the potential of this approach; further optimization of clinical compliant methods for genetic modifications of NK cells is needed to exploit the full clinical potential of this approach. Moreover, additional research on the multiple aspects of NK cell tumor targeting that could be modified with this technique is needed. Although clinical responses following infusion of NK cells may be further improved by simply augmenting their tumor targeting capacity, studies evaluating the potential of this technology to improve the persistence of infused cells as well as avenues to promote proper NK cell migration and homing to the tumor tissue are also warranted (Figure 1).

Genetic engineering of NK cells to make them cytokine independent and thereby improve persistence, while boosting their cytotoxic capacity, may be one avenue to further explore. The advantage with this approach would be that exogenous cytokines would be unnecessary following NK cell infusion, which may reduce the risk of mobilizing regulatory T cells that directly suppress NK cell cytotoxicity (13). Challenges with taking this approach to a clinical context include the risk of inducing a cytokine release syndrome due to massive and unregulated NK cell proliferation. This approach also comes with the potential risk of inducing malignant transformation of the NK cells due to permanent autocrine growth stimulation, as have been observed for IL-2 engineered T cells (72). However, such scenarios may be avoided if genes coding for IL-2 or IL-15 are only temporarily introduced via mRNA electroporation of NK cells. Should stable transgene expression be required to induce proper tumor regression, an alternative strategy to prevent runaway NK cell proliferation would be to introduce an inducible suicide gene in the modified cells (73).

Migration to the tumor tissue is another aspect governing proper tumor targeting. This aspect has been largely overlooked and could potentially improve clinical outcome if infused NK cells are redirected to the tumor site instead of circulating non-specifically into mostly non-tumor-bearing tissues. No studies aimed at improving the *in vivo* homing of infused gene engineered NK cells have yet been published.

As discussed above, numerous strategies for redirecting or boosting NK cell tumor killing *in vitro* have been explored. Introduction of CARs represent the most studied and developed approach that has recently reached clinical evaluation (Table 2). Expression of the high-affinity CD16 may soon also be tested in a clinical setting as this approach can be combined with already clinically available monoclonal antibodies that target an array of antigens expressed on a variety of different tumor types. Bolstering NK cell cytotoxicity via autocrine cytokine stimulation or via silencing of inhibitory NK cell receptors will likely require additional evaluation in preclinical animal models before they can be incorporated in clinical protocols. Once all these strategies are fully characterized pre-clinically, they may be combined to further improve the full anti-tumor potential of adoptively transferred NK cells. For instance, introduction of a CAR while simultaneously silencing the NKG2A inhibitory receptor may represent one such future approach. One can also consider adding autocrine cytokine stimulation to further improve cytotoxicity while simultaneously supporting their *in vivo* persistence. As NK cell degranulation is regulated by a balance of activating and inhibitory signals from well-defined cell surface receptors, it may also be possible to add CARs or other activation receptors together with selected receptors that mediate inhibition via ligands that are expressed on normal tissues (and not tumor cells), thereby giving genetically reprogramed NK cells an additional layer of target specificity. However, many additional preclinical studies will be needed before these approaches can reach clinic.

The choice of method for genetic reprograming of NK cells is another important factor that needs to be considered when taking genetically engineered NK cells to clinical evaluation. Viral transduction has the advantage of stable expression; however, as mentioned above, viral transduction of NK cells, especially primary cells, does not always lead to a satisfactory level of transgene expression and may require multiple rounds of transduction followed by selection of transgene positive cells. Moreover, proper expression of transgenes induced by viral transduction can take days, which may be of disadvantage since the lifespan of an NK cell may be relatively short following adoptive transfer (i.e., weeks). Future studies are warranted to better understand if multiple infusions of transfected NK cells can compensate transient transgene expression or if stable transgene expression is a prerequisite for inducing clinical responses following adoptive transfer of genetically engineered NK cells. Studies are also needed to fully understand the lifespan of NK cells, particularly those that have undergone *ex vivo* manipulation.

The optimal method for genetic manipulation of NK cells to be used in a clinical trial may also depend on what NK cell preparation is used (Box 3). The advantage with NK cell lines is that they can be utilized as an off-the-shelf product stably transduced to express the gene or genes of interest. They may also be long-lived if given the proper cytokine support. However, the downside with using NK cell lines, like NK-92, is the requirement for irradiation (10 Gy) prior to infusion to avoid the risk of engrafting cells that are potentially tumorigenic *in vivo* (74). Moreover, patients treated with infusions of NK cell lines would also need moderate to high level of preconditioning to suppress host immunity to avoid rejection of these allogeneic cells. Moreover, infusion of allogeneic cells can raise humoral immunity and lead to adaptive T-cell immune responses specifically against alloantigens, precluding repeated infusions even with the use of preconditioning. Similar allo-reactivity can be induced with the use of primary allogeneic NK cell infusions. The use of autologous NK cells circumvents these risks and precludes the need for preconditioning. The potential draw back with using autologous NK cells is that efficient tumor targeting can be prevented by inhibitory KIR interactions with self-HLA. A potential advantage with using an NK cell line versus primary NK cells is that large numbers of NK cells from the NK cell line can be infused, whereas the number of primary cells available for infusion are typically much more limited. However, this limitation has recently been circumvented by a number of highly efficient methods to expand primary NK cells *ex vivo* for clinical infusion (60). Ideally, infusion of autologous gene-modified NK cells can be used to avoid the rejection risk and the prerequisite for preconditioning. One approach to overcome limitations of autologous NK cell inactivation via self-HLA is to genetically modify these effectors to silence inhibitory self-HLA binding receptors, such as NKG2A and KIRs, which alone or in combination with for instance CARs, can improve the tumor targeting capacity of NK cells in the autologous setting.

Box 3 **NK Cell Source for Adoptive NK Cell Cancer Immunotherapy.**

**Table:** 

NK cell source	Pros	Cons
NK cell lines (NK-92)	Readily available	Preconditioning needed
	Easy to gene manipulate	Host immunity against alloantigens limits repeated infusions and *in vivo* persistence?
Primary non-expanded NK cells	Autologous cells, no rejection. No need for cell expansion *ex vivo*	Low number
Primary *ex vivo* expanded NK cells	High numbers of highly activated autologous cells available for repeated use	GMP laboratory needed for expansion
		Costs for reagents

## Concluding Remarks

Anti-tumor antibodies and CAR T cells have established immunotherapy as a viable treatment option for patients with cancer. Given their rapid and efficient method of recognizing tumor cells, NK cells represent a unique immune cell to genetically reprogram in an effort to improve the outcome of cell-based cancer immunotherapy. However, technical and biological challenges associated with gene delivery into NK cells have significantly tempered this approach. Viral transduction of NK cells initially resulted in low transgene delivery efficiencies that often required multiple rounds of transduction and/or cellular enrichment to achieve acceptable numbers of transgene expressing cells. Nevertheless, recent improvements in retro- and lentiviral transduction of NK cells have led to a flurry of preclinical studies on gene engineered NK cells. A number of studies have also shown that NK cells can be genetically reprogramed using mRNA electroporation. In contrast to viral transduction, this approach offers high transfection efficiencies without compromising their viability and does not require high-level biosafety laboratories. Although promising preclinical data on mRNA electroporated NK cells have emerged recently, concerns have been raised regarding the clinical utility of this approach as it only results in transient transgene expression.

Recently initiated clinical trials will soon give insight into the potential effectiveness of cell-based cancer immunotherapy strategies that utilize genetically modified NK cells. Nevertheless, further optimization of both viral transduction and electroporation of NK cells is still needed before this approach can be fully exploited in the clinic. With the recent advances in our understanding of the complex biological networks that regulate the capacity of NK cells to target and kill tumors *in vivo*, and with rapid developments in clinically compliant techniques to genetically manipulate NK cells, we foresee genetic engineering as an obligatory pathway to exploit the full potential of adoptive NK cell immunotherapy in patients with cancer.

## Conflict of Interest Statement

The authors declare that the research was conducted in the absence of any commercial or financial relationships that could be construed as a potential conflict of interest.
